# Computed tomography coronary angiography accuracy in women and men at low to intermediate risk of coronary artery disease

**DOI:** 10.1007/s00330-012-2503-5

**Published:** 2012-06-06

**Authors:** Anoeshka S. Dharampal, Stella L. Papadopoulou, Alexia Rossi, Annick C. Weustink, Nico R. A. Mollet, W. Bob Meijboom, Lisan A. Neefjes, Koen Nieman, Eric Boersma, Pim J. de Feijter, Gabriel P. Krestin

**Affiliations:** 1 Department of Radiology, Erasmus MC, Room Ca207a, ‘s-Gravendijkwal 230, P.O. Box 2040, 3015 CE Rotterdam, The Netherlands; 2 Department of Cardiology, Erasmus MC, Rotterdam, The Netherlands

**Keywords:** Diagnostic accuracy, CT coronary angiography, Multidetector computed tomography, Coronary artery disease, Duke pre-test probability, Sex, women and men

## Abstract

**Objectives:**

To investigate the diagnostic accuracy of CT coronary angiography (CTCA) in women at low to intermediate pre-test probability of coronary artery disease (CAD) compared with men.

**Methods:**

In this retrospective study we included symptomatic patients with low to intermediate risk who underwent both invasive coronary angiography and CTCA. Exclusion criteria were previous revascularisation or myocardial infarction. The pre-test probability of CAD was estimated using the Duke risk score. Thresholds of less than 30 % and 30–90 % were used for determining low and intermediate risk, respectively. The diagnostic accuracy of CTCA in detecting obstructive CAD (≥50 % lumen diameter narrowing) was calculated on patient level. *P* < 0.05 was considered significant.

**Results:**

A total of 570 patients (46 % women [262/570]) were included and stratified as low (women 73 % [80/109]) and intermediate risk (women 39 % [182/461]). Sensitivity, specificity, PPV and NPV were not significantly different in and between women and men at low and intermediate risk. For women vs. men at low risk they were 97 % vs. 100 %, 79 % vs. 90 %, 80 % vs. 80 % and 97 % vs. 100 %, respectively. For intermediate risk they were 99 % vs. 99 %, 72 % vs. 83 %, 88 % vs. 93 % and 98 % vs. 99 %, respectively.

**Conclusion:**

CTCA has similar diagnostic accuracy in women and men at low and intermediate risk.

***Key Points*:**

• *Coronary artery disease (CAD) is increasingly investigated by computed tomography angiography (CTCA)*.

• *CAD detection or exclusion by CTCA is not different between sexes*.

• *CTCA diagnostic accuracy was similar between low and intermediate risk sex-specific-groups*.

• *CTCA rarely misses obstructive CAD in low–intermediate risk women and men*.

• *CAD yield by invasive coronary angiography after positive CTCA is similar between sex-risk-specific groups*.

## Introduction

Cardiovascular disease is the leading cause of death in the western world [1]. For several years awareness has been growing that cardiovascular disease is also the primary cause of death in women not only in the western world but also in economic developing countries [2]. Part of this high mortality is related to under-recognition, underdiagnosis and undertreatment of coronary artery disease (CAD) in women [3–7].

The interpretation of chest pain in women caused by CAD is often difficult because of the “less typical” symptoms in women compared with the classical “typical” symptoms in men [3, 4, 8]. This may cause a delay or lead to incorrect diagnosis [3, 4]. Additionally first-line non-invasive diagnostic tests such as exercise electrocardiography and single photon emission computed tomography (SPECT) imaging in women are less sensitive and specific [5, 6].

Paradoxically, because of the diagnostic uncertainty there appears to be an overuse of invasive coronary angiography (ICA) in women, with a rather low diagnostic yield of obstructive CAD [9, 10]. This prompted a “red alert call” for the promotion of advanced non-invasive imaging techniques in women including CT coronary angiography (CTCA) [11]. CTCA has evolved as a reliable gatekeeper of ICA because negative CT findings virtually rule out the presence of obstructive CAD [12–14]. Whether this also applies to women remains unresolved because women have been under-represented in studies assessing the diagnostic accuracy of CTCA [12–17]. The pre-test probability (PTP) of obstructive CAD plays a significant role in the assessment of the clinical utility of CTCA. This PTP of obstructive CAD can be derived from reported clinical prediction rules [18] and may influence the post-CTCA probability of obstructive CAD and thus the need for further testing and management.

The purpose of our study was to assess the diagnostic accuracy and clinical utility of CTCA to detect or exclude obstructive CAD in women with low to intermediate PTP of obstructive CAD in comparison to men.

## Methods and materials

### Study population

Patients referred by their treating physician for ICA between July 2004 and June 2009 on the basis of chest pain presentation (typical, atypical and non-anginal complaints) with or without outcome of stress testing were invited to undergo CTCA within 2 weeks of ICA. The CTCA outcome did not affect referral to ICA. We excluded patients with known iodine allergy, impaired renal function and patients with previous revascularisation. A total of 907 patients gave consent for CT accuracy studies and were entered in our single-centre registry. Out of these 907 patients we excluded patients with previous myocardial infarct (*n* = 54) as well as patients with a high PTP (>90 %) of having obstructive CAD (*n* = 283). The final study sample comprised a total of 570 symptomatic patients. The study was approved by the institutional review board of our medical hospital.

### Computed tomography imaging protocol and image reconstruction

Patients underwent single-source CT (Somatom Sensation, Siemens Healthcare, Forchheim, Germany) between 2004 and 2006 and subsequently underwent dual-source CT (Somatom Definition, Siemens Healthcare). The imaging settings are described in Table 1. Heart-rate-lowering medication (Metoprolol, Seloken, Astra Zeneca, Zoetermeer, Netherlands) was administered when the heart rate was above 65 beats per minute before the single-source CT in the absence of contraindications to achieve better quality images. To achieve better visualisation of the small coronary arteries the vasodilating agent Nitrolingual (Nitroglycerin Pumpspray, G.Pohl-boskamp, Itohenlockstedt, Germany) was administered before imaging in the absence of contraindications.

**Table 1:** Imaging protocol

	SSCT^b^	DSCT^c^
β-blocker^a^	Yes	No
Nitroglycerin^a^	No	Yes
X-ray tube	1	2
Collimation	32 × 0.6 (Z-FFS)	32 × 0.6 (Z-FFS)
Gantry rotation time (ms)	330	330
Temporal resolution [ms]	165	83
Spatial resolution (mm)	0.4 × 0.4 × 0.4	0.4 × 0.4 × 0.4
Pitch	0.2	0.2–0.53
Rotation time (ms)	330	330
Unenhanced imaging		
Tube voltage (kV)	120	120
Tube current	200–150 effective mAs	75 mAs/rotation
ECG-triggered tube current modulation	Yes	Yes
Contrast-enhanced imaging		
Tube voltage (kV)	120	120
Tube current	850–960 effective mAs	320–412 mAs/rotation
ECG-triggered tube current modulation	No	Yes

*SSCT* single-source CT, *DSCT* dual-source CT, *Z-FFS* Z-flying focal spot, *ECG* electrocardiogram, *mAs/rotation* total mA × rotation time

^a^Administration before imaging when no contraindication was present

^b^Somatom Sensation, Siemens Healthcare, Forchheim, Germany

^c^Somatom Definition, Siemens Healthcare, Forchheim, Germany

All patients initially underwent unenhanced CT to calculate the calcium score using the Agatston method [19]. Subsequently a bolus tracking technique was used to synchronise the start of image acquisition with the arrival of the iodinated contrast agent [Iomeprol, iomeron (400 milligram iodine per millilitre), Bracco, Milan, Italy; Ultravist, iopromide (370 milligram iodine per millilitre), Schering Berlin, Germany] in the coronary arteries followed by a saline chaser.

Between 2004 and 2006 a full-dose ECG-synchronised spiral imaging technique was used. After the introduction of ECG-triggered tube current modulation, the spiral imaging technique with ECG pulsing was used in the subsequent years to reduce the effective radiation dose. Data sets were reconstructed retrospectively according to the heart rate to obtain motion-free images. For heart rates of at least 80 beats per minute the data sets were reconstructed in 31–47 % of the R–R interval (systolic phases). For heart rates of no greater than 65 beats per minute this value was 60–76 % of the R–R interval (diastolic phases). For heart rates between 66 and 79 beats per minute reconstructions were needed in both systolic and diastolic phases, 30–77 % of the R–R interval. Images were analysed using medium-to-smooth convolution kernels for non-calcified lesions and sharp convolution kernels for calcified lesions.

### Computed tomography image evaluation

All data sets were transferred for analyses to an offline proprietary workstation (Leonardo, Siemens, Erlangen, Germany); the total calcium score was calculated using dedicated proprietary software (CaScoring). Two experienced observers with more than 2 years of experience in cardiac CT, blinded to the ICA results, independently evaluated all CTCAs for the presence of CAD, using axial source images, multiplanar, curved reformatted reconstructions, and thin-slab maximum intensity projections using “Circulation” software on the proprietary workstation. Interobserver disagreements were resolved by a joint consensus reading.

The modified 17-segment American Heart Association model was used to classify each segment [20]. Each segment was visually scored as obstructive in the presence of at least 50 % lumen diameter narrowing and non-obstructive when the lumen diameter narrowing was less than 50 % in comparison with the proximal and distal lumen. All anatomically available coronary segments with a diameter of at least 1.5 mm, irrespective of image quality or calcification, were included and scored with the intention to diagnose. Un-evaluable segments of poor quality due to calcification, stack, motion artefacts, or low contrast enhancement were scored as obstructive. Coronary segments distal to a total occlusion could not be optimally visualised by ICA and thus were excluded from the analyses. The image quality was scored on a segment level as diagnostic and non-diagnostic. The average of this score per patient was compared between women and men.

### Invasive coronary angiogram image evaluation

One experienced cardiologist, blinded to the CT results, visually assessed each coronary segment (American Heart Association model) for the presence of luminal narrowing in two orthogonal planes. Stenoses scored as having more than 20 % narrowing on visual assessment were quantified using the validated quantitative coronary angiography (QCA) algorithm (CAASII [Cardiovascular Angiography Analysis System II]; Pie Medical Imaging Maastricht, the Netherlands). The segments were considered obstructive when the quantified lumen diameter narrowing in one of the two planes was at least 50 %. Obstructive lesions on a segment level were used to determine obstructive CAD on a patient level as having one or more obstructive stenoses (≥50 % lumen diameter stenosis) irrespective of the segment in which they were located.

### PTP of obstructive CAD

We used the Duke risk score [18] to estimate the PTP of obstructive CAD due to the presence of multiple risk factors such as age, gender, symptoms, history of myocardial infarction, ECG, smoking, hypercholesterolaemia and diabetes mellitus. We used less than 30 %, 30–90 %, and greater than 90 % as thresholds for the low, intermediate and high risk group, respectively. As patients with a high risk are directly referred for ICA we did not include these patients in our analyses [21, 22].

The observed PTP of obstructive CAD is the prevalence of obstructive CAD defined as having at least one lesion with at least 50 % lumen diameter stenosis per patient detected by ICA.

### Statistical analyses

The statistical analysis was performed using a dedicated statistical software program (SPSS, version 16.0, IMB, Chicago, IL, USA). Categorical variables were expressed as percentages and continuous variables were expressed as means ± standard deviation. Continuous variables with a skewed distribution were expressed as median with interquartile range.

The estimated PTP was compared in women, men and in the different risk groups with the observed PTP (observed prevalence) using the paired *t* test.

The patients’ characteristics were compared between women and men and between different risk groups using the independent *t* test; otherwise the Mann-Whitney *U* test was used (skewed distribution of continuous data). The interobserver variability was tested between the two CT readers on segment and patient level using the *κ* statistic.

The CTCA results were compared with the reference standard ICA on a patient level to calculate the sensitivity (SN), specificity (SP), positive predictive value (PPV) and negative predictive value (NPV) [23]. The Wilson score [24] was used to calculate the confidence intervals for small groups. The diagnostic accuracy and utility were compared across the different risk groups, between women and men using the chi-squared test or the Fisher’s exact test in the presence of less than five observations in a cell of the 2 × 2 table. A *P* value of less than 0.05 was considered statistically significant.

The receiver-operating characteristic (ROC) curve was used for visual analyses of the trade-offs between the sensitivity and the specificity of CTCA. The area under the ROC curve (AUC) was calculated to account for the influence of referral bias and prevalence of disease on the diagnostic accuracy of CTCA in our population [25, 26].

The clinical utility of CTCA was a measure of diagnostic certainty or remaining uncertainty of absence or presence of CAD that is derived from the post-test probability of CAD. In case of diagnostic certainty no further testing is required, whereas remaining uncertainty requires further diagnostic testing. It was assumed that a post-test probability of obstructive CAD of less than 5% indicated high certainty with no further requirement for diagnostic testing; probabilities between 5 and 90 % represented uncertainty and indicated a requirement for further diagnostic testing; and probabilities of greater than 90 % indicated high certainty with direct referral to ICA, as proposed by previous studies [27].

This study was performed according to the criteria set forth in the Standard for Reporting of Diagnostic Accuracy Initiative [28].

## Results

In total, 570 patients at low to intermediate risk were included of which 46 % were women (*n* = 262). The patients’ characteristics are provided according to sex and risk group in Table 2. Women overall had a significantly higher heart rate (HR) than men (*P* = 0.03). The HR was similar in the different risk groups (Table 2). The prevalence of CAD (at least one obstructive lesion) in women was 61 % and in men this was 64 % (*P* = 0.42). The PTP of obstructive CAD determined by the Duke risk score (estimated PTP) was significantly lower than the observed prevalence of obstructive CAD determined by ICA in women in the overall group (45 % vs. 61 %, *P* < 0.01) as well as in the low (17 % vs. 48 %, *P* < 0.01) and intermediate risk groups (60 % vs. 66 %, *P* = 0.02) separately. In men there was no difference between the estimated PTP and observed prevalence (overall 65 % vs. 64 %, *P* = 0.56; low 21 % vs. 28 %, *P* = 0.43; intermediate risk group (70 % vs. 68 %, *P* = 0.38).

**Table 2:** Patients’ characteristics

	All				Low				Intermediate			
	Women		Men		Women		Men		Women		Men	
	Mean	[SD]	Mean	[SD]	Mean	[SD]	Mean	[SD]	Mean	[SD]	Mean	[SD]
Number patients	262		308		80		29		182		279	
Age (years)	62*	**[10.96]**	55*	**[9.52]**	54*	**[8.58]**	40*	**[8.15]**	66*	**[9.57]**	57*	**[8.18]**
Chest pain complaints^a^												
Typical	30 %* (78)	***[0.46]***	20 %* (63)	***[0.40]***	1 %* (1)	***[0.11]***	0 %* (0)	***[0.00]***	42 %* (77)	***[0.50]***	23 %* (63)	***[0.42]***
Atypical	38 %* (100)	***[0.49]***	50 %* (155)	***[0.50]***	49 %* (39)	***[0.50]***	41 %* (12)	***[0.50]***	34 %* (61)	***[0.47]***	51 %* (143)	***[0.50]***
Non-anginal	32 % (84)	[0.47]	29 % (90)	[0.46]	50 % (40)	[0.50]	59 % (17)	[0.50]	24 % (44)	[0.43]	26 % (73)	[0.44]
Current smoker	23 % (59)	[0.42]	28 % (86)	[0.45]	14 % (11)	[0.34]	10 % (3)	[0.31]	26 % (48)	[0.44]	29 % (83)	[0.46]
Diabetes mellitus^b^	16 % (43)	[0.37]	12 % (36)	[0.33]	4 % (3)	[0.19]	0 % (0)	[0.00]	22 %* (40)	***[0.42]***	13 %* (36)	***[0.34]***
CAD in family^c^	55 %* (143)	***[0.50]***	44 %* (137)	***[0.50]***	59 % (47)	[0.50]	41 % (12)	[0.50]	53 % (96)	[0.50]	45 % (125)	[0.50]
Hypercholesterolaemia^d^	56 %* (147)	***[0.50]***	44 %* (135)	***[0.50]***	41 %* (35)	***[0.50]***	10 %* (3)	***[0.31]***	62 %* (112)	***[0.49]***	47 %* (132)	***[0.50]***
Hypertension^e^	52 %* (138)	***[0.50]***	40 %* (122)	***[0.49]***	48 %* (38)	***[0.50]***	10 %* (3)	***[0.31]***	54 %* (100)	***[0.50]***	42 %* (119)	***[0.50]***
Body mass index (kg/m^2^)	27	[4.45]	27	[3.51]	27	[4.62]	26	[2.56]	27	[4.38]	27	[3.59]
Calcium score^f^	139*	***[5–446]***	349*	***[9–401]***	20	[0–194]	2	[0–65]	211	[36–613]	155	[14–444]
Prevalence CAD segments/patient^g^	9 %	[0.11]	10 %	[0.12]	7 %*	***[0.09]***	3 %*	***[0.06]***	10 %	[0.11]	11 %	[0.13]
One vessel disease	35 % (92)	[0.48]	32 % (98)	[0.47]	33 % (26)	[0.47]	21 % (6)	[0.41]	36 % (66)	[0.48]	33 % (92)	[0.47]
Multi-vessel disease^h^	25 % (65)	[0.43]	32 % (99)	[0.47]	14 % (11)	[0.35]	7 % (2)	[0.26]	30 % (54)	[0.46]	35 % (97)	[0.48]
Estimated PTP^i^	45 %*	**[0.25]**	65 %*	**[0.21]**	17 %*	**[0.07]**	21 %*	**[0.08]**	60 %*	**[0.18]**	70 %*	**[0.17]**
Observed PTP^j^	61 % (159)	[0.49]	64 % (197)	[0.48]	48 % (38)	[0.50]	28 % (8)	[0.45]	66 % (121)	[0.47]	68 % (189)	[0.47]
HR (beats per minute)	67*	**[11.94]**	65*	**[12.64]**	67	[11.91]	65	[13.78]	67	[11.98]	65	[12.54]
Radiation exposure (mSv)^k^	12	[3.78]	12	[4.05]	12	[3.24]	13	[3.56]	12	[3.92]	12	[4.09]

Data in parentheses are number of patients. Data in brackets are the standard deviations and for the calcium score the 25th and 75th percentiles

*Bold values represent significant values (*P* < 0.05) in the comparison between women and men using Student’s* t* test or Mann–Whitney *U* test (italics)

^a^We defined typical angina as substernal discomfort that was precipitated by physical exertion or emotion and relieved by rest or nitroglycerin within 10 min. We classified chest pain with only 1 or 2 of these 3 symptom characteristics as atypical angina pectoris; if none of the characteristics was present, we classified it as non-anginal chest pain

^b^Treatment with oral antidiabetic medication or insulin

^c^Patient had first- or second-degree relatives with premature CAD

^d^Total cholesterol >180 mg/dl or treatment for hypercholesterolaemia

^e^Blood pressure >120/90 mmHg or treatment for hypertension

^f^Agatston score with median and [interquartile range]

^g^[(Number diseased segments × 100%)/(total of number segments)] per patient

^h^>1 vessel with obstructive CAD detected by ICA

^i^Estimated probability of obstructive CAD [18]: low, <30 % (estimated PTP); intermediate, 30–90 % (estimated PTP)

^j^Prevalence of obstructive CAD on a patient level: observed probability of obstructive CAD determined by ICA

^k^mSv (millisievert): dose length product × 0.017

In this population the prevalence of obstructive CAD and extent of CAD in terms of multi-vessel disease and calcium score were similar between women and men (Table 2).

The interobserver variability for detection of obstructive stenosis showed a good agreement on segment (*κ* = 0.94 [95 % CI 0.93–0.95]) and patient levels (*κ* = 0.91 [95 % CI 0.87–0.95]). The diagnostic image quality was not significantly different between women and men (0.96 vs. 0.97, *P* = 0.06).

### Diagnostic accuracy of CTCA

The patient-based diagnostic accuracy of CTCA for detecting or ruling out obstructive CAD according to ICA revealed an AUC of 0.895 (95 % CI: 0.862 - 0.928) which was similar between women and men (0.867 [95 % CI: 0.815 - 0.920] vs. 0.921 [95 % CI: 0.880 - 0.961], *P* = 0.06), with similar sensitivity and specificity for women and men when grouped in the low and intermediate risk groups and across the low and intermediate risk groups in both women and men (Table 3).

**Fig. 1:**
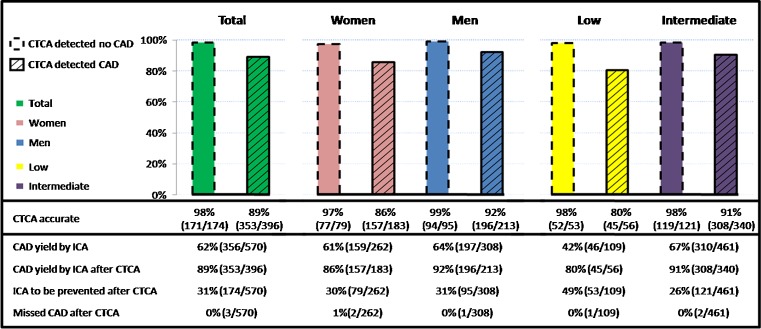
CAD detection by CTCA and its influence on the yield of CAD by ICA

**Table 3:** Diagnostic accuracy and clinical utility of CTCA between women and men across different risk groups

Risk groups		Women	Men		Women				Men				*P* value (W–M)
		*n*	*n*		SN	SP	PPV	NPV	SN	SP	PPV	NPV	
All	TP	157	196	SN	99 [96–100]				99 [97–100]				0.59*
	TN	77	94	SP		77 [66–82]				85 [77–90]			0.07
	FP	26	17	PPV			86 [80–90]				92 [88–95]		0.047
	FN	2	1	NPV				97 [91–99]				99 [94–100]	0.59*
Low	TP	37	8	SN	97 [87–100]				100 [68–100]				1.00*
	TN	33	19	SP		79 [64–88]				90 [71–97]			0.31*
	FP	9	2	PPV			80 [67–89]				80 [49–94]		1.00*
	FN	1	0	NPV				97 [85–99]				100 [83–100]	1.00*
Intermediate	TP	120	188	SN	99 [95–100]				99 [97–100]				1.00*
	TN	44	75	SP		72 [60–82]				83 [74–90]			0.10
	FP	17	15	PPV			88 [81–92]				93 [88–96]		0.12
	FN	1	1	NP				98 [88–100]				99 [93–100]	1.00*
*P* value (L–I)					0.42*	0.46	0.23	1.00*	1.00*	0.52*	0.19*	1.00*	

The diagnostic accuracy (SN, SP) and clinical utility (PPV, NPV) values are expressed as percentages with [95 % confidence interval]. Estimated PTP [18] <30 % was defined as low and 30–90 % as belonging to the intermediate risk group. *P* value L–I (low vs. intermediate risk group) and W–M (women vs. men) estimated using chi-squared test and (*) Fisher’s exact test

*TP* true positive, *TN* true negative, *FP* false positive, *FN* false negative, *SN* sensitivity, *SP* specificity, *PPV* positive predictive value, *NPV* negative predictive value

Because different CT systems were used over the years we also assessed the diagnostic performance for detecting CAD between these two machines. No significant differences were found between the single-source and dual-source CT systems for sensitivity (99 % vs. 99 %, *P* = 0.93), specificity (84 % vs. 75 %, *P* = 0.10), positive predictive value (85 % vs. 91 %, *P* = 0.06) and negative predictive value (99 % vs. 97 %, *P* = 0.39).

### Clinical utility of CTCA

Certainty defined as probabilities for obstructive CAD of no greater than 5 % after negative CTCA was achieved in both women (NPV 97 % and 98 % for low and intermediate risk, respectively) and men (NPV 100 % and 99 % for low and intermediate risk, respectively) at low to intermediate risk. No certainty of obstructive CAD after positive CTCA could be achieved (probability <90 %) in women at low (PPV 80 %) and intermediate risk (PPV 88 %), and men at low risk (PPV 80 %) (Table 3).

### Yield of obstructive CAD by ICA after CTCA

A negative CTCA would prevent 31 % (174/570) of the low to intermediate risk patients proceeding to ICA (30 % (79/262) women; 31 % (95/308) men, *P* = 0.86; Fig. 1). This prevention of ICA would be more pronounced in patients at low risk (low 49 % (53/109) vs. intermediate 26 % (121/461), *P* < 0.001) compared to patients at intermediate risk. Obstructive CAD would be missed in less than 1 % after a negative CTCA with no significant differences between women and men (1 % (2/262) women vs. 0 % (1/308) men, *P* = 0.48), or between the low and intermediate risk groups (1 % (1/109) low vs. 0 % (2/461) intermediate risk group, *P* = 0.52). A positive CTCA would result in 89 % (353/396) yield of obstructive CAD by ICA with no significant differences between women and men (86 % (157/183) women vs. 92 % (196/213) men, *P* = 0.0502) or between the low and intermediate risk groups (80 % (45/56) low vs. 91 % (308/340) intermediate risk group, *P* = 0.06).

## Discussion

In our clinical evaluation of CTCA in low to intermediate risk women and men with suspected CAD who were referred for ICA but underwent additional CTCA we found that:

- The Duke risk score underestimated the prevalence of angiographically obstructive CAD in women, especially in those at low risk;
- The sensitivity and specificity of CTCA were similar across low and intermediate risk groups in and between women and men;
- The clinical utility of a positive CTCA was moderate, with remaining diagnostic uncertainty in low to intermediate risk women and low risk men requiring additional functional testing;
- The yield of obstructive CAD by ICA was similar in women and men with low and intermediate risk;
- CTCA was highly accurate in excluding the presence of obstructive CAD in women and men at low and intermediate risk.

In our study the overall predicted probability of having obstructive CAD using the Duke risk score was underestimated in women (45 % vs. 61 %, *P* < 0.01) compared with the actual presence of CAD assessed by ICA. This finding probably reflects the difficulties of evaluating women with symptoms suspected of CAD and has been described in previous studies [3, 8]. Physicians may misclassify the symptoms as non-anginal or atypical angina in women, which consequently affects the Duke risk score where symptom presentation plays a significant role in the calculation of the PTP. Underestimation of the Duke risk score may also be related to the referral bias of our study population, in particular in women who were referred by their treating physicians for ICA.

It is noteworthy that the diagnostic accuracy of CTCA in detecting obstructive CAD was similar in women and men (Table 3; sensitivity 99 % vs. 99 %; specificity 77 % vs. 85 % for women and men, respectively). Similar findings were shown by Meijboom et al. in a multicentre study (sensitivity 100 % vs. 99 %; specificity 63 % vs. 66 %) [14] and by Pundziute et al. in a single-centre study (sensitivity 95 % vs.100 %; specificity 93 % vs. 89 % for women and men, respectively) [29]. This is in contrast to the reported technical limitations and diminished accuracy in women of other non-invasive ischaemia tests [6, 30] and underscores the gender neutrality (equality) of the diagnostic accuracy of CTCA.

The clinical utility of CTCA, i.e. reflecting the remaining diagnostic certainty of the presence or absence of significant CAD, is an important criterion in the overall clinical assessment of CTCA. The clinical utility is derived from the post-test probability of CAD and even with the same diagnostic accuracy of a test (sensitivity/specificity) it is different in patients with low or intermediate PTP of CAD. We arbitrarily assumed that in patients with a post-test probability of less than 5 % or greater than 90 % sufficient diagnostic certainty was achieved and no further diagnostic testing was required. In patients with a post-test probability between 5 % and 90 % diagnostic certainty was not sufficient and in these patients further diagnostic testing was deemed necessary. In our population we found that the clinical utility of a positive CTCA was high (>90 %) in men at intermediate PTP of CAD and further diagnostic testing was not necessary. These patients may then be referred to ICA. The clinical utility of a positive CTCA was moderate in women at low to intermediate PTP and in men of low PTP of CAD. In these patients diagnostic certainty was insufficient and further diagnostic testing may be needed. Higher certainty may be then achieved by functional testing for ischaemia e.g. by SPECT or by stress echocardiography [31, 32].

The clinical utility of a negative CTCA was high in women and men at low or intermediate PTP of CAD providing high certainty (<5 %) of absence of obstructive CAD, and no further testing would be necessary. It has been shown in numerous studies that the absence of CAD detected by CTCA is associated with an excellent prognosis [33, 34], which further lends support that these patients may be safely discharged. It should be noted that in patients with persistent chest complaints due to microvascular dysfunction, normal CT findings with no obstructive epicardial CAD may be seen [35]. In these patients, who are often women, further diagnostic testing for ischaemia to detect microvascular dysfunction will be needed to adjust clinical management and survival [36].

Recent studies reported that the yield of elective ICA to demonstrate the presence of obstructive CAD in women and men is 27–49 % and 47–67 %, respectively, in patients referred for ICA [9, 10], whereas by performing CTCA before diagnostic ICA the yield may improve to 86 % for women and 92 % for men (Fig. 1) and may decrease the number of unnecessary ICA in women and men by 30 % and 31 %, respectively, as suggested by our study.

Our study has limitations. Referral bias was present, because the patients in our population were referred by their treating physicians to undergo invasive coronary imaging on the basis of their chest pain presentation with or without outcome of stress testing. This may explain the relatively high prevalence of obstructive CAD, in particular in women. To account for the influence of referral bias on the diagnostic accuracy [25, 26] of CTCA we also reported the AUC which was not different between women and men (0.87 vs. 0.92, *P* = 0.056). Our study population only consisted of stable patients and our results may not apply to the wider spectrum of patients with suspected CAD who did not undergo ICA or who have unstable symptoms. Of concern was the rather high radiation exposure of CTCA (12 mSv) due to the use of a retrospective imaging protocol, which was standard in first-generation CT [14, 37]. Currently with the newer-generation systems and optimal imaging protocols much lower levels of radiation exposure (<3 mSv) can be achieved in patients with low heart rates (<65 beats per minute) [38].

In conclusion, computed tomography coronary angiography has similar diagnostic accuracy in women and men with low and intermediate risk, and may function as an efficient gatekeeper for ICA in women as well as in men.

## Abbreviations

AUC: area under receiver-operating characteristic curve
CAD: coronary artery disease
CTCA: computed tomography coronary angiography
ECG: electrocardiography
HR: heart rate
ICA: invasive coronary angiography
NPV: negative predictive value
PPV: positive predictive value
PTP: pre-test probability
SN: sensitivity
SP: specificity
SPECT: single photon emission computed tomography
QCA: quantitative coronary angiography
